# The transversus abdominis plane block may reduce early postoperative pain after laparoscopic ventral hernia repair a matched pair analysis

**DOI:** 10.1016/j.amsu.2020.05.044

**Published:** 2020-06-08

**Authors:** C. Paasch, N. Aljedani, P. Ortiz, S. Azarhoush, J. Fiebelkorn, K.A. Boettge, U. Gauger, S. Anders, G. De Santo, M.W. Strik

**Affiliations:** a Department of General, Visceral and Cancer Surgery, Helios Klinikum Berlin-Buch, Schwanebecker Chaussee 50, 13125, Berlin, Germany; b No Insurance Surgery, 9121 W Russell Rd Ste 115, Las Vegas, 89148, USA; c U. Gauger, Berlin, Germany

**Keywords:** Transversus abdominis plane, TAP-Block, IPOM, Ventral hernia repair

## Abstract

**Purpose:**

Patients suffering from a ventral hernia can be treated by laparoscopic ventral hernia repair (VHR) with the intraperitoneal onlay mesh (IPOM) technique. To reduce early postoperative pain and the analgesic cumulative need for medication (CNM), the transversus abdominis plane (TAP) block has recently been investigated and implemented in hernia surgery. We aimed to investigate its impact when conducting a VHR in IPOM technique.

**Methods:**

A single center retrospective observational matched pair analysis has been conducted from March to April 2020. The data of patients who underwent VHR in IPOM technique with prior TAP block administration were enrolled. The matching was performed using the variables age ( ±5 years), gender, type of surgery, BMI and ASA stage.

**Results:**

52 patients were enrolled. Among the individuals of the TAP block group, (18 males, 8 females) the average age was 52.4 (15.9). The average BMI was 29.0 (3.95) kg/m^2^. 14 patients suffered from an umbilical, 9 from an incisional, and three from an epigastric hernia. Except for COX-2-inhibitors, (TAP group: 41.9 mg (31.0), Control group 9.23 (22.1), p < 0.001) the analgesic CNM of both groups did not statistically differ from each other. The literature review yielded four relevant publications (n = 100). The authors stated a positive impact of the TAP block on early postoperative pain and analgesic medication consumption.

**Conclusion:**

The TAP block prior to laparoscopic ventral hernia repair may reduce early postoperative pain and analgesic medication consumption in selected patients. More randomized clinical trials are needed to confirm these findings.

## Highlights

• The transversus abdominis plane block may reduce early postoperative pain after laparoscopic ventral hernia repair.
• Four relevant publication (n = 100) has been yielded on that topic.
• Review findings are contradictorily.

## 1 Background

Ventral hernia repair is one of the most commonly performed surgical procedures. In 2006, approximately 348.000 ventral hernias were operated on in the United States of America. This led to a total cost of 3.2 billion US-dollars [1,2].

In general, a tailored approach is advisable for VHR [[3], [4], [5]]. Besides other surgical approaches, the laparoscopic IPOM placement has been described as a sufficient approach for VHR [6,7].

Frequently, patients are suffering from postoperative acute and chronic pain following VHR in IPOM technique [7]. In particular, the use of sutures and staples for mesh fixation has been discussed as being reasonable [8].

To reduce postoperative acute and chronic pain and to accelerate early recovery, the effect of the transversus abdominis plane (TAP) block has recently been investigated in hernia repair [9]. Most of these publications focused on inguinal hernia repair [10]. However, the TAP block has also been conducted prior to VHR. Feierman et al. (2014) observed a sufficient analgesic effect of the TAP block for 13 patients who underwent open umbilical hernia repair [11]. In addition, a prospective randomized clinical trial among 70 individuals who underwent VHR revealed pain reduction through a TAP block [12].

The evidence in literature regarding the effectiveness of the TAP block prior to VHR in laparoscopic IPOM technique remains low. Our review yielded only three relevant publications. In all cases, (n = 48) the TAP block has been done via ultrasound guidance and showed a pain reduction [[13], [14], [15]].

In order to reveal the impact of the TAP block on early postoperative pain after laparoscopic ventral hernia repair, we conducted the matched pair analysis and the review of literature at hand.

## 2 Methods

A monocentric retrospective observational matched pair analysis investigating the efficacy of the TAP block prior to VHR in IPOM technique was performed. The patients’ data from June 2014 to April 2020 who underwent surgery with this technique were taken from their files.

The study occurred at the Helios Hospital Berlin-Buchxxx xxxx (Germany) between March and April 2020. Due to the anonymous study setting a consent form was not needed. The study was approved by the Ethics Committee of the ‘Ärztekammer Berlinxx’ (Medical Association Berlinxx) in March 2020 (Eth-06/20) and conducted in accordance with the ethical standards of the Helsinki Declaration 1975.

The study was registered with the German clinical trial registry DRKS (DRKS00021122). No funding has been received. The trial is based on the patients’ data available from their files. The time of their hospital stay has been analyzed. A systematic follow-up has not been performed.

The study has been reported in line with the STROCSS criteria [16].

### 2.1 Inclusion criteria

Patients who underwent elective and emergency VHR in laparoscopic IPOM technique were enrolled.

### 2.2 Exclusion criteria

Individuals who underwent a conversion to an open VHR were excluded.

### 2.3 TAP block technique

The TAP block was conducted under direct visualization with the laparoscope in the beginning of the operation. With the abdomen insufflated, the operating surgeon palpated the lateral border of the rectus sheath to ensure adequate lateral placement. Following, a 19G needle was inserted percutaneously just above the iliac spine and inferior to the costal margin, as lateral as possible within the surgical field. Using the laparoscope to ensure no penetration of the peritoneum, the needle was advanced through the internal and external obliques. Then, a small amount of anesthetic was injected into both TAPs. The dispersal of the anesthetic along the TAP was confirmed visually prior to the injection of the entire amount.

The TAP block consisted of 0.375% (20 ml) ropivacaine (RV).

### 2.4 Surgical procedure

The surgical approach was conducted under general anesthesia. A single shot of antibiotics was administrated for incisional hernia repairs.

We performed a subcostal incision within the left abdominal wall to insert the optical trocar after gas insufflation via Veress needle. Additionally, we placed, visual-guided, two 5-mm trocars within the left abdomen. After thorough adhesiolysis, we removed the hernial sac. The hernial orifice was closed using a non-resorbable suture, which was placed using a Reverdin needle. The polyester mesh, (Composite mesh, Medtronic©) along with multiple sutures, was inserted into the abdominal cavity and laid out covering the weak spot. The mesh was fixated to the abdominal wall with resorbable tacks.

### 2.5 Statistical analysis

Analysis was done using R (ver. 3.6.1). Data were presented as number (percentages) for nominal or mean ± SD/median (min-max) for metric variables. For the comparison of nominal variables between groups, Fishers exact test was used. Metric variables normality were tested by using the Shapiro-Wilk test. The T-test or the Wilcoxon-test were used in order to assess significant differences. A p-value < 0.05 was considered statistically significant. No corrections for multiple tests were done.

Matching was done in the following manner: According to patients age ( ±5 years), gender, BMI (WHO classification [17]) and type of surgery, the data of the control group were enrolled.

Due to the composition of the study pool, a matching with the ASA score was not possible. “ASA I patients” were allocated to “ASA I + II patients”, “ASA II patients” to “ASA II + III patients” and “ASA III patients” to “ASA II + III patients”.

### 2.6 Aims

The primary endpoint was the CNM of opioids on the day of operation.

The secondary endpoint was the evaluation of pain after surgery in the PACU using VAS (visual analog scale). The patients were asked once about their pain while in the recovery room. In some cases, the patients were asked several times. The mean value was calculated in these cases.

Additional secondary endpoints were CNM of non-opioid agents on the day of operation, relevant complications according to clavien-dindo-classification during the hospital stay (CDC [18]), operating time and length of hospital stay (LOS).

As standard analgesic medication, all patients received ibuprofen 2400 mg/d during their hospital stay or metamizole 4 g/d. Depending on the treating medical staff, COX-2-inhibitors, morphine, oxycodone, acetaminophen, piritramide or metamizole have been given as needed in the PACU.

The premedication was given by the anesthesiologist depending on the patients physical and mental comorbidities. It consisted of oxycodone, COX-2-inhibitors, or no medication. For the statistical analysis the premedication agents were added to the CNM.

### 2.7 Database

In March 2019, a MS Excel data sheet was provided. The data was imported into R (ver. 3.6.1), and multiple plausibility checks were conducted. In April 2020, updates of the data were provided while inconsistencies were resolved.

### 2.8 Review of literature

We reviewed the literature using Pubmed and Google Scholar (Table 4). The following search terms were used: “Ventral”, “Hernia”, “IPOM”, “Epigastric”, “Transversus”, “Abdominis”, “plane”, “TAP block”.

## 3 Results

### 3.1 Baseline characteristics

The TAP block group consisted of 26 patients. The average age was 52.4 (15.9). In this study, 18 individuals were male and 8 patients female. The average BMI was 29.0 (3.95) kg/m^2^. Regarding the ASA stage, 8 individuals had an ASA score of I and 10 patients an ASA score of II, while in 8 cases an ASA score of III was revealed. No patient had an ASA score of IV. 14 patients suffered from an umbilical, 9 from incisional, and 3 patients from epigastric hernias (Table 1). 24 individuals received a visual-guided and two an ultrasound-guided TAP block.

**Table 1:** Basic data and patient characteristics for surgical subgroups.

Variable		Control group	TAP group	*p*-value
		*n = 26*	*n = 26*	
Age		53.0 (14.8)	52.4 (15.9)	0.886
Sex	*male*	18 (69.2%)	18 (69.2%)	1.000
	*female*	8 (30.8%)	8 (30.8%)	
ASA preoperative	*I*	11 (42.3%)	8 (30.8%)	0.107
	*II*	13 (50.0%)	10 (38.5%)	
	*III*	2 (7.69%)	8 (30.8%)	
	*IV + V*	0 (0%)	0 (0%)	
Body Mass Index (BMI)	kg/m2	29.2 (3.15)	29.0 (3.95)	0.838

ASA = American Society of Anesthesiologists physical status classification.

Continuous measurements are presented as mean (SD); TAP transversus abdomins plane.

### 3.2 Univariate analysis on baseline characteristics

26 of the 52 participating patients were assigned to the group “TAP block” and 26 individuals to the control group. Table 1 shows the univariate analysis on baseline characteristics. No significant differences were observed.

### 3.3 Univariate analysis on endpoints

#### 3.3.1 Primary endpoint: CNM of opioids

Patients in the TAP-Block group received an average of 4.77 mg (13.00) of morphine and oxycodone on the day of their operation. Individuals of the control group received an average of 8.85 mg (6.26) of morphine and oxycodone on the day of their operation (p = 0.158; Table 3).

Patients in the TAP-Block group received an average of 2.46 mg (3.31) of piritramide on the day of their operation. Individuals in the control group received an average of 1.35 mg (3.26) of piritramide on the day of their operation (p = 0.227; Table 3).

#### 3.3.2 Secondary endpoint: LOS

The average LOS was 2.23 days (0.51) for the TAP-Block group, and 3.35 days (2.78) for the control group (p = 0.055; Table 2).

**Table 2:** Perioperative data for surgical groups.

Variable		Control group	TAP group	*p*-value
		*n = 26*	*n = 26*	
Type of hernia	*EH*	3 (11.5%)	3 (11.5%)	1.000
	*ICH*	*9* (34.6%)	9 (34.6%)	
	*UH*	14 (53.8%)	14 (53.8%)	
Operating time	*minutes*	48.1 (19.6)	44.8 (15.2)	0.495
CDC Grading	*0*	22 (84.6%)	25 (96.2%)	0.110
	*I*	4 (15.4%)	0 (0%)	
	*II*	0 (0%)	0 (0%)	
	*III*	0 (0%)	1 (3.85%)	
	*IV + V*	0 (0%)	0 (0%)	
LOS	*days*	3.35 (2.78)	2.23 (0.51)	0.055

CDC Clavien-dindo classification; Continuous measurements are presented as mean (SD); EH epigastric hernia; IH incisional hernia.

TAP transversus abdominis plane; UH umbilical hernia.

#### 3.3.3 Secondary endpoint: Operating time

The average operating time was 44.8 min (15.2) for the TAP-Block group, and 48.1 min (19.6) for the control group (p = 0.495; Table 2).

#### 3.3.4 Secondary endpoint: CNM of acetaminophen, metamizole and nonsteroidal anti-inflammatory drugs (NSAID), COX-2-inhibitors

Patients in the TAP-Block group received an average of 0.46 g (1.79) of acetaminophen on the day of their operation. Individuals of the control group received no acetaminophen on the day of their operation (p = 0.202).

Patients in the TAP-Block group received an average of 8.88 g (10) of metamizole on the day of their operation. Individuals of the control group received an average of 10.9 g (11.4) of metamizole on the day of their operation (p = 0.505).

Patients in the TAP-Block group received an average of 33.7 g (113) of NSAIDs on the day of their operation. Individuals of the control group received an average of 32.0 g (111) of NSAIDs on the day of their operation (p = 0.958) (Table 3).

**Table 3:** Univariate analysis on pain and cumulative need medication.

Variable		Control group	TAP group	*p*-value
		*n = 26*	*n = 26*	
Pain level in PACU	*VAS-Score*	1.09 (1.28)	1.04 (1.16)	0.884
CNM of acetaminophen	*g*	0.00 (0)	0.46 (1.79)	0.202
CNM of metamizol	*g*	10.9 (11.4)	8.88 (10.0)	0.505
CNM of morphin and oxycodon	*mg*	8.85 (6.26)	4.77 (13.0)	0.158
CNM of piritramid	*mg*	1.35 (3.26)	2.46 (3.31)	0.227
CNM of NSAIDs	mg	32.0 (111)	33.7 (113)	0.958
CNM of COX-2-Inhibitor	mg	9.23 (22.1)	41.9 (31.0)	<0.001
No CNM required		26 (100%)	26 (100%)	1.000

CNM Cumulative need medication; Continuous measurements are presented as mean (SD).

NSAID Nonsteroidal anti-inflammatory drugPACU Postanaesthetic care unit.

TAP Transversus abdominis plane; VAS Visual analog scale.

**Table 4:** Publications on TAP block administration in laparoscopic IPOM VHR.

Authors/Year/Country	Study design	Sample size *n*	Type of TAP block administration	Exclusion criteria	TAP block impact
Fields et al., /2015/USA	RCT	100 (52 TAP-Block)	VG, intraoperative, BV 0.25% (50 ml)	Conversion to open Surgery	No significant difference in pain scores at 24 h postoperatively; reduced analgesic CNM
Sinha et al., /2018/India	Double blind RCT	30 (15 TAP block)	UG, postoperativly, RV 0.375% (20 ml)	>ASA II stage	Reduced early postoperative pain (12 h*)
Jain et al., /2019/India	RCT	50 (25 TAP block)	UG, preoperativly + port site infiltration RV 0.5% (10 ml)	>ASA II stage	Reduced early postoperative pain + CNM (12 h*)
Bhatia et al., /2019/India	Case series	8	UG, preoperatively RV 0,5% (5 ml)	>ASA II stage, large hernias, IH, UH	Median VAS score < 3 in 6/8 patients
Own results/2020/German	Matched pair analysis	52 (26 TAP block)	VG, intraoperative, RV 0.375% (20 ml)	–	No impact* on pain + analgesic CNM

BV bupivacaine; CNM cumulative need medication; IH incisional hernia.

PACU Postanaesthetic care unit; RV ropivacaine; TAP Transversus abdominis plane.

UG ultrasound-guided; UH umbilical hernia; VG visual guided; VHR Ventral hernia repair.

* Statistical significant.

Patients in the TAP-Block group received an average of 41.9 g (31.0) of COX-2-Inhibitors on the day of their operation. Individuals of the control group received an average of 9.23 g (22.1) of COX-2-Inhibitors on the day of their operation (p = 0.958) (Table 3).

#### 3.3.5 Secondary endpoint: pain after surgery in the PACU on the day of operation

Using a VAS score, patients of the TAP-Block group stated an average pain level of 1.04 (1.16) in the recovery room. Individuals in the control group stated an average pain level of 1.09 (1.28) in the recovery room (p = 0.884; Table 3).

#### 3.3.6 Secondary endpoint: Postoperative complications

25 patients (92.15%) in the TAP-Block group had no complications (CDC = 0). One patient (3.8%) had to be re-operated on due to intraabdominal bleeding.

22 patients (84.61%) in the control group had no complications. Four individuals (15.4%) suffered from a CDC = 1 complication (hypokalaemia, urinary infection, urinary retention). No individual from the control group had a CDC=III complication (Table 3). The differences do not reach statistical significance (p = 0.110).

### 3.4 Review of literature

The search yielded four publications containing 100 patients (not applied in 40 cases, 30 women, 30 men). Table 4 depicts the details of literature review.

## 4 Discussion

The impact of the TAP block on postoperative pain and analgesic medication administration in VHR using the IPOM technique has recently been investigated. Our review of literature yielded four relevant publications (n = 100; Table 4). Fields et al. (2015) performed a RCT among 100 patients. 52 individuals received a visual-guided TAP block with 0.25% bupivacaine (50 ml). Patients with higher morbidity were not excluded. Both groups were similar in terms of biometric and perioperative data. The pain level was measured one and 24 h after surgery. The authors did not reveal a significant difference but the patients in the TAP block group needed less analgesic CNM. Sinha et al. (2018) conducted a randomized double-blind prospective study (n = 30). All patients underwent laparoscopic VHR. 15 patients were allocated to the TAP block group with RV (ultrasound-guided and conducted after surgery). 15 individuals received saline solution through the TAP block. The patients receiving the TAP block with RV experienced significant less pain in the PACU. Gender distribution was not stated. The authors excluded patients of higher morbidity such as coronary artery disease, heart block and bradycardia [15]. Jain et al. (2019) analyzed 50 patients who underwent laparoscopic uncomplicated ventral hernia repair using the IPOM technique. In 25 cases, an ultrasound-guided TAP block was conducted which resulted in a decreased VAS score (0,5, 1, 1,5, 2, 4, 6, 12 and 24 h postoperatively) and decreased administration of rescue analgesics [14]. Gender distribution was not stated. All 30 patients received port site infiltration with RV. Only patients with low morbidity (ASA I + II) were enrolled. When conducting an ultrasound-guided TAP block among 8 patients prior to laparoscopic epigastric hernia repair in IPOM technique, Bhatia et al. (2019) observed a median VAS score below 3 in 75% of cases (n = 6). The authors include only ASA I + II patients with small hernias and did not provide a matched control group [13].

Summarized, one publication did not detect an impact of the TAP block on early postoperative pain. Three publications revealed that the ultrasound-guided TAP block positively affected the pain sensation and the analgesic CNM requirement. The authors conducted the VHR in IPOM technique but only in selected patients (ASA I + II). When conducting the matched pair analysis at hand (n = 56), like Fields et al. (2015) we did not reveal the positive impact of the TAP block administration on early postoperative pain sensation, it could be explained by the fact that we did not exclude patients with a high morbidity. 30.7% (8/26) had an ASA-score of III in the TAP block group. The morbidity may consist of patients suffering from chronic pain. However, when excluding ASA III patients in our analysis, we did not reveal any differences in terms of pain in the PACU and analgesic medication administration. Additionally, there is an assumption that the TAP block location was conducted falsely, a more proximal approach may be more efficient. It could lead to a reduced effect of the analgesic on the cranial part of the fixated mesh. It is possible that the tacks are causing pain, but some published trials contradict this assumption [19,20]. Another explanation may be different concentrations of the administered RV (Table 4). We administrated 0.375% RV in 20 ml. In comparison, Sinha et al. (2018) injected the same doses when conducting their double blind RCT while showing a positive impact on early postoperative pain [15]. However, higher doses were injected by Jain and Bhatia et al. [13,14]. They also detected a pain reduction of the TAP block group. Moreover, it is imaginable that the positive impact of the TAP block reflects the superiority of the ultrasound-guided administration compared to the visual-guided administration. In our study, the majority of 24 cases received a visual-guided and two individuals an ultrasound-guided TAP block. As a random sample, we analyzed these two matched pairs and did not reveal any significant differences regarding the pain level and administration of analgesic CNM. In addition, Fields et al. (2015) did not detect significant differences in terms of the pain level 24 h after surgery and these authors, like mentioned, also conducted the TAP block visual-guided [21].

In conclusion, all yielded trials and the study at hand are difficult to compare due to different agents, concentrations of RV, time and approach of its administration (preoperatively, postoperatively and intraoperatively; ultrasound-guided, visual-guided).

However, the prospective trials revealed a positive impact and the findings of a double blinded RCT [15]are more evident than those of a matched-pair analysis, such as the one at hand. Including our results, there are only 126 patients described who underwent VHR in IPOM technique and received a TAP block (Table 4). Therefore, it remains uncertain whether the TAP block administration leads to less pain and a reduced analgesic need after VHR IPOM technique. As a result of the increasing evidence of the TAP block effectiveness following inguinal hernia repair [9]. Its administration should be considered. Nevertheless, rare complications such as liver trauma and femoral nerve palsy should not be forgotten [22,23]Further prospective clinical trials are needed.

Individuals, who received the TAP block in our study, needed less oxycodone (8.85 mg (6.26) vs. 4.77 mg (13.0) TAP group, statistically not significant p = 0.158) but more COX-2-Inhibitors (9.23 mg (22.1) vs. 41.9 mg (31.0) TAP group, p < 0.001; Table 3). One explanation might be the fact that we recently started to implement COX-2-Inhibitors in cases of required premedication.

The evaluation of pain after surgery in the PACU was a secondary endpoint. In this study the visual analog scale (VAS) was used. In future studies different methods of pain measurement e.g. Numeric Rating Scale and Verbal Rating Scale should also be compared [24]. Moreover when conducting randomized trials on that topic, it seems to be mandatory to measure the pain at set times after surgery. Until now, only the ultrasound-guided TAP block in VHR using the IPOM technique has been investigated [[13], [14], [15]]. Most of our patients (n = 24) received a visual-guided TAP block. Further trials should compare both approaches.

The relatively small sample size and the retrospective study design are study limitations. Regarding matching pairs with their ASA-score, we were unable to find suitable pairs. Therefore, “ASA I patients” were allocated to “ASA I + II patients”, “ASA II patients” to “ASA II + III patients” and “ASA III patients” to “ASA II + III patients”. The TAP block group consisted of 8 patients with an ASA score of III whereas only two patients had a higher morbidity in the control group. Moreover, we did not routinely measure the pain level in the PACU.

## 5 Conclusions

The TAP block prior to laparoscopic ventral hernia repair may reduce early postoperative pain and analgesic medication administration in selected patients. More randomized clinical trials are needed to confirm these findings.

## Ethical approval

The study was approved by the Ethics Committee of the ‘Ärztekammer Berlin’ (Medical Association Berlin) in March 2020 (Eth-06/20) and conducted in accordance with the ethical standards of the Helsinki Declaration 1975.

## Funding

All authors have no source of funding.

## Author contribution

Dr. med. Christoph Paasch (corresponding author):

Contribution to the paper: author, data collection, data analysis and interpretation, writing the paper, examination and treatment of the patient.

Mrs. Nouf Aljedani (co-author).

Contribution to the paper: data extraction.

Mr. Pedro Ortiz (co-author).

Contribution to the paper: data extraction.

Fr. Jennifer Fiebelkorn (co-author):

Contribution to the paper: data extraction.

Dr. Sascha Azarhoush (co-author):

Contribution to the paper: data extraction.

Dr. Gianluca De Santo (co-author):

Contribution to the paper: data analysis, examination and treatment of the patient.

Fr. Katherina Böttge (co-author):

Contribution to the paper: data analysis, examination and treatment of the patient.

Dr. Ulrich Gauger (co-author):

Contribution to the paper: data analysis.

Dr. med. Stefan Anders (co-author):

Contribution to the paper: data analysis, examination and treatment of the patient.

Prof. Dr. Martin W. Strik (co-author):

Contribution to the paper: data analysis, examination and surgical treatment of the patient.

## Registration unique identifying number (UIN)

The study was registered with the German clinical trial registry DRKS (DRKS00021122). No funding has been received.

https://www.drks.de/drks_web/setLocale_EN.do.

## Guarantor

Dr. med. Christoph Paasch.

## Provenance and peer review

Not commissioned, externally peer reviewed.

## Declaration of competing interest

None.
